# *Actinobacillus pleuropneumoniae* challenge in swine: diagnostic of lung alterations by infrared thermography

**DOI:** 10.1186/s12917-014-0199-2

**Published:** 2014-09-16

**Authors:** Anne Menzel, Martin Beyerbach, Carsten Siewert, Melanie Gundlach, Doris Hoeltig, Robert Graage, Hermann Seifert, Karl-Heinz Waldmann, Jutta Verspohl, Isabel Hennig-Pauka

**Affiliations:** Clinic for Swine and Small Ruminants, Forensic Medicine and Ambulatory Services, University of Veterinary Medicine Hannover, Bischofsholer Damm 15Aff105, D-30173 Hannover, Germany; Institute for Biometry, Epidemiology and Information Processing, University of Veterinary Medicine, Hannover, Germany; Institute for General Radiology and Medical Physics, University of Veterinary Medicine, Hannover, Germany; Clinic for Swine, University of Veterinary Medicine, Vienna, Austria; Institute for Microbiology, University of Veterinary Medicine, Hannover, Germany

**Keywords:** Infrared thermography, Imaging techniques, Computed tomography, Aerosol challenge, Diagnostic method, Skin surface temperature, Regional temperature differences, Respiratory tract disease

## Abstract

**Background:**

*Actinobacillus pleuropneumoniae (A.pp.)* is the causative agent of porcine pleuropneumonia leading to high economic losses in the pig industry. Infrared thermography (IRT) of the thorax might offer a new method to select swine with lung alterations for further diagnostics.

In this study 50 german landrace pigs were infected with *A.pp.* in an established model for respiratory tract disease, while 10 healthy pigs served as control animals. To avoid drift errors during IR measurements absolute skin temperatures and temperature differences between a thoracal and an abdominal region were assessed for its diagnostic validity.

**Results:**

IRT findings during the course of experimental *A.pp.-*infection were verified by computed tomography (CT) before and on days 4 and 21 after infection. Significant correlations were found between clinical scores, CT score and lung lesion score. Ambient temperature, body temperature and abdominal surface temperature were factors influencing the skin surface temperature of the thorax. On day 4 but not on day 21 after infection the right thoracal temperature was significantly higher and the difference between a thoracal region in the height of the left 10th vertebra and an abdominal region was significantly lower in infected pigs than in control pigs. At a cut off of 28°C of right thoracal temperature the specificity of the method was 100% (CI 95%: 69-100%) and the sensitivity 66% (CI 95%: 51-79%).

At a cut off of 2°C temperature difference between thoracal and abdominal region on the left body site the specificity of the method was 100% (CI 95%: 69-100%) and the sensitivity 32% (CI 95%: 19-47%) with all control pigs detected negative.

Orientation for lung biopsy by IRT resulted in 100% specificity and sensitivity (CI 95%: 69-100%) of bacteriological examination of tissue samples during the acute stage of infection.

**Conclusion:**

IRT might be a valuable tool for the detection of inflammatory lung alterations in pigs, especially during the acute stage of infection and if ambient temperatures are constant during individual measurements. External and internal factors interfere with this method, so that its application in the field might be restricted to a selection of pigs for further diagnostic with adequate specificity.

## Background

Porcine respiratory tract diseases lead to high economic losses in the swine industry worldwide. Clinical signs of disease can be reduced by vaccination against porcine respiratory tract pathogens, but under field conditions the protective effect is often limited in the case of multifactorial disease with several pathogens involved. In general, all vaccines available failed to prevent infection and transmission of pathogens [1,2]. In general, after the onset of acute respiratory disease on a farm, selected pigs are euthanized and necropsied to initiate bacteriological and virological examination of lung tissue. In addition, serological tests are performed in living pigs, which give information about contact with specific pathogens in the past, but not if lung tissue is affected or not [3].

Subclinically and chronically diseased animals do not show any clinical symptoms [4]; consequently, they cannot be selected efficiently for further diagnostic procedures or sorted out prior to slaughter. Therefore one clinical research aim in the field of diagnostic of respiratory diseases in pigs is to identify pigs with lung alterations which do not show clinical signs, to select them for further diagnostic procedures and by this increase the chance of a successful diagnosis as a basis for solving the herd problem.

The practicability of imaging techniques of the lung like digital radiography and computed tomography (CT) is restricted by high personal and investigation costs [5,6]. Ultrasonography was found to be more convenient but was of lower sensitivity, because lung lesions covered by aerated lung tissue were undetectable [7-9].

Infrared thermography (IRT) as an imaging method for the detection of elevated body temperature, circulatory disorders or circumscribed organ inflammation would be of high advantage in swine medicine because of its fast, cheap and non-invasive applicability.

One approach to overcome measurement inaccuracies due to a time-dependent drift error of the IR camera is the calculation of a difference temperature between two defined body localizations as an allocation base [10]. In the current study, temperature differences between regions of interest (ROI) were used to overcome IRT device errors and variations between different IRT images. Selected anatomical localizations could be reproducibly found in the IRT image. As depicted in the manufacturer’s instruction of the used IR camera measurement accuracy was ± 1.5°C which is comparable to other IR cameras used in medicine diagnostics [11].

Dependence on ambient temperature is one major cause for the limited practical use of IRT in medicine up to now. In several studies in veterinary medicine IRT was tested as a diagnostic tool, e.g. to detect a pneumothorax in rats [12] or spine diseases and neuromuscular disorders in horses [13,14]. In calves, the skin temperature around the eyes was found to be a sensitive marker to detect changes in thermoregulation due to respiratory disease [15].

In pigs, IRT failed as a diagnostic tool for arthritis, because thick subcutaneous fatty tissue layers prevented the detection of a local inflammatory temperature increase [16].

In swine no reference values for thoracal skin temperatures in pigs with inflammatory lung alterations exist up to now. In the following study, a systematic approach was chosen to evaluate IRT as a diagnostic tool for the detection of changes in the surface temperature pattern of the thorax after infection with the lung pathogen *Actinobacillus pleuropneumoniae* (*A.pp.).*

The aim of this study was to examine the applicability of passive infrared thermography for the diagnostic of lung tissue alterations in pigs at different stages of infection. Clinical examination and CT were used to assess the lung health status during the experiment as an allocation base for IRT. Comparison between both imaging techniques resulted in the determination of specific regions of interest (ROI) for temperature measurements. At the end of the study, IRT findings were compared to findings from gross pathology and histological examinations.

## Results

### Animal model: clinical and post-mortem scores

Prior to infection all animals were tested negative for *A.pp.* in an Apx-II-Enzyme-linked immunosorbant assays (ELISA) and were defined as healthy according to a clinical score (CLS) and a CT score (CTS). After infection, challenged pigs developed respiratory disease of varying severity reflected by increased CLS, CTS, lung lesion scores (LLS) and scores according to S.P.E.S. (Table 1). A significant correlation was found between the S.P.E.S. score and the LLS (r_sp_ = 0.72, p < 0.01).

**Table 1:** **Clinical, computed tomographic and post-mortem scores in infected and control pigs**

	**Prior to infection (n = 50)**	**Day 4 (n = 47, CT score n = 46)**	**Day 21 (n = 40, CT score n = 39)**
**Clinical score**			
Mean ± σ	**0.00** ± 0.02	**4.68** ± 3.86	**25.16** ± 33.13
Median	0.00	3.82	8.22
Range	0.00 – 0.13	1.16 – 17.63	4.52 – 92.63
**CT score**			
Mean ± σ	**0.03** ± 0.09	**5.39** ± 1.50	**2.11** ± 1.80
Median	0.00	5.25	1.63
Range	0.00 – 0.47	0.79 – 7.71	0.00 - 7.31
**Lung lesion score** [30] **of infected pigs** (n = 50)			
Mean ± σ			**12.45** ± 7.656
Median			11.7
Range			1.58 – 33.68
**S.P.E.S. score** [32] **of infected pigs** (n = 42)			
Median			4
75th percentil			4
Range			1-4
**Lung lesion score** [30] **of control pigs** (n = 10)			
Mean ± σ			**0.11** ± 0.33
Median			0
Range			0 – 1.05
**S.P.E.S. score** [32] **of control pigs** (n = 10)			
Median			0
75th percentil			0
Range			0-1

Several animals showed typical signs of acute pleuropneumonia, as elevated body temperature up to 41.8°C, dyspnoea, coughing, apathy and vomiting.

Twelve pigs had to be euthanized before the end of the experiment, because they suffered from generalized systemic disease with severe dyspnoea. They underwent CT and infrared thermographical examination prior to gross pathological examination.

The acute stage of infection was characterized by disseminated lung tissue alterations recorded by CT.

On day 21 after infection significant correlations were found between CLS, CTS and LLS (CLS-LLS: r_p_ = 0.45, p = 0.01; CTS-LLS: r_p_ = 0.81, p < 0.01 CLS - CTS: r_p_ = 0.68, p < 0.01). Gross pathological findings ranged from normal lung tissue to severe pleuropneumoniae.

Pigs of the control group stayed healthy until the end of the experiment.

### Surface skin temperatures measured by infrared thermography (IRT) during experimental infection and influencing factors

Data of ten control pigs and of all infected pigs were analyzed for surface skin temperature changes during the course of infection.

Multiple regression analysis revealed a variety of external and internal factors influencing the diagnostic command variables absolute surface temperatures of the thorax as well as skin surface temperature differences between thoracal and abdominal region (Tables 2 and 3). Most important influencing variables were ambient temperature, body core temperature and abdominal skin temperature.

**Table 2:** **Correlations between dependent and independent variables on day 4 after infection**

**Influencing variables**	**Dependent variables**							
	**Pearsson’s correlation coefficient, p- (probability) value, number of pigs**							
	**Temperature difference between thoracal and abdominal region on the left body site**		**Temperature difference between thoracal and abdominal region on the right body site**		**Left thoracal temperature**		**Right thoracal temperature**	
	**Infected**	**Control**	**Infected**	**Control**	**Infected**	**Control**	**Infected**	**Control**
Age (living days)	0.07	0.42	−0.04	*0.72*	−0.08	−0.38	−0.03	−0.33
	0.63	0.23	0.78	*0.02*	0.58	0.28	0.85	0.36
	47	10	47	10	47	10	47	10
Body weight (kg)	−0.01	0.52	0.01	0.50	*0.33*	−0.18	*0.35*	0.05
	0.96	0.12	0.93	0.14	*0.02*	0.62	*0.01*	0.89
	47	10	47	10	47	10	47	10
Clinical score	0.06	0.28	−0.18	0.43	*−0.54*	−0.26	*−* *0.51*	0.06
	0.67	0.43	0.22	0.22	*<0.01*	0.46	*<0.01*	0.87
	47	10	47	10	47	10	47	10
Computed tomography score	−0.13	0.41	*−0.30*	0.47	−0.12	0.18	−0.12	−0.23
	0.40	0.23	*0.04*	0.17	0.43	0.62	0.42	0.53
	46	10	46	10	46	10	46	10
Breathing frequency (per minute)	−0.11	−0.08	−0.05	−0.20	0.29	0.44	0.27	0.14
	0.45	0.82	0.76	0.58	0.05	0.21	0.07	0.70
	46	10	46	10	46	10	46	10
Heart rate (per minute)	0.09	0.32	−0.03	0.09	0.07	*−* *0.65*	0.08	−0.45
	0.54	0.36	0.86	0.81	0.64	*0.04*	0.60	0.19
	46	10	46	10	46	10	46	10
Body temperature (rectal)	*0.34*	−0.35	*0.38*	−0.37	*0.30*	*0.83*	0.18	*0.82*
	*0.02*	0.33	*<0.01*	0.29	*0.05*	*<0.01*	0.22	*<0.01*
	46	10	46	10	46	10	46	10
Ambient temperature (°C)	0.03	−0.53	−0.00	−0.21	*0.75*	0.31	*0.78*	*0.64*
	0.89	0.11	0.98	0.56	*<0.01*	0.38	*<0.01*	*0.04*
	46	10	46	10	46	10	46	10
Abdominal temperature (left,°C)	−0.01	−0.21	*0.31*	−0.28	*0.96*	*0.75*	*0.94*	*0.89*
	0.93	0.56	*0.04*	0.44	*<0.01*	*0.01*	*<0.01*	*<0.01*
	47	10	47	10	47	10	47	10
Abdominal temperature (right,°C)	−0.10	−0.20	*0.31*	−0.04	*0.93*	0.58	*0.94*	*0.94*
	0.52	0.58	*0.04*	0.92	*<0.01*	0.08	*<0.01*	*<0.01*
	47	10	47	10	47	10	47	10
Lung lesion score	−0.21	−0.43	*−* *0.31*	−0.45	−0.11	0.51	−0.11	0.38
	0.15	0.22	*0.04*	0.19	0.48	0.13	0.48	0.27

Surface skin temperatures as well as temperature differences between thoracal and abdominal regions on the left and right body sites were influenced by several internal and external factors. Pearsson’s Correlation Coefficients (upper lines) between dependent and influencing variables, their levels of significance (middle lines) and the number of evaluated pigs (lower line) are shown. From 50 infected pigs three pigs had died prior to day 4 after infection.

**Table 3:** **Correlations between dependent and independent variables on day 21 after infection**

**Influencing variables**	**Dependent variables**							
	**Pearsson´s correlation coefficient, p- (probability) value, number of pigs**							
	**Temperature difference between thoracal and abdominal region on the left body site**		**Temperature difference between thoracal and abdominal region on the right body site**		**Left thoracal temperature**		**Right thoracal temperature**	
	**Infected**	**Control**	**Infected**	**Control**	**Infected**	**Control**	**Infected**	**Control**
Age (living days)	−0.085	0.13	0.07	−0.03	0.13	0.36	0.18	0.44
	0.60	0.72	0.67	0.93	0.44	0.31	0.29	0.20
	40	10	40	10	40	10	40	10
Body weight (kg)	−0.04	−0.29	0.15	−0.33	*0.36*	*0.73*	*0.36*	*0.72*
	0.83	0.41	0.36	0.35	*0.02*	*0.02*	*0.02*	*0.02*
	40	10	40	10	40	10	40	10
Clinical score	−0.07	−0.30	−0.04	−0.18	−0.09	0.40	0.02	0.31
	0.68	0.34	0.82	0.63	0.58	0.25	0.91	0.38
	40	10	40	10	40	10	40	10
Computed tomography score	−0.17	0.17	−0.11	0.14	−0.07	0.13	−0.04	0.12
	0.30	0.65	0.50	0.69	0.68	0.72	0.81	0.74
	39	10	39	10	39	10	39	10
Breathing frequency (per minute)	0.09	0.28	0.04	0.56	0.23	−0.13	0.16	−0.22
	0.59	0.43	0.83	0.09	0.16	0.72	0.33	0.53
	40	10	40	10	40	10	40	10
Heart rate (per minute)	−0.02	0.18	0.00	0.41	*0.36*	−0.25	*0.44*	−0.30
	0.91	0.62	0.99	0.24	*0.02*	0.49	*<0.01*	0.40
	40	10	40	10	40	10	40	10
Body temperature (rectal)	0.28	0.15	*0.43*	−0.01	*0.64*	*0.67*	*0.72*	*0.73*
	0.08	0.68	*<0.01*	0.98	*<0.01*	*0.04*	*<0.01*	*0.02*
	40	10	40	10	40	10	40	10
Ambient temperature (°C)	−0.24	0.02	0.13	0.07	*0.72*	0.07	*0.59*	0.09
	0.14	0.96	0.43	0.86	*<0.01*	0.85	*<0.01*	0.80
	40	10	40	10	40	10	40	10
Abdominal temperature (left,°C)	0.08	0.42	*0.38*	0.33	*0.95*	*0.642*	*0.87*	*0.69*
	0.61	0.23	*0.02*	0.35	*<0.01*	*0.04*	*<0.01*	*0.03*
	40	10	40	10	40	10	40	10
Abdominal temperature (right,°C)	0.05	−0.03	*0.47*	0.02	*0.88*	*0.91*	*0.92*	*0.92*
	0.74	0.93	*<0.01*	0.97	*<0.01*	*<0.01*	*<0.01*	*<0.01*
	40	10	40	10	40	10	40	10
Lung lesion score	−0.20	−0.50	−0.09	−0.14	0.05	−0.12	0.10	−0.20
	0.21	0.14	0.57	0.70	0.76	0.75	0.54	0.57
	40	10	40	10	40	10	40	10

Surface skin temperatures as well as temperature differences between thoracal and abdominal regions on the left and right body sites were influenced by several internal and external factors. Pearsson´s Correlation Coefficients (upper lines) between dependent and influencing variables, their levels of significance (middle lines) and the number of evaluated pigs (lower line) are shown.

Because individual pigs were examined subsequently by IRT imaging in a cooled chamber on fixed examination days, ambient temperature increased in parallel to the duration of examination due to the presence of the warm, living pigs and the examiner. The high impact also of a relatively cold ambient temperature (6-15°C) onto sensitivity and specificity of the method is reflected in the results of IRT measurements in this trial.

**Figure 1:**
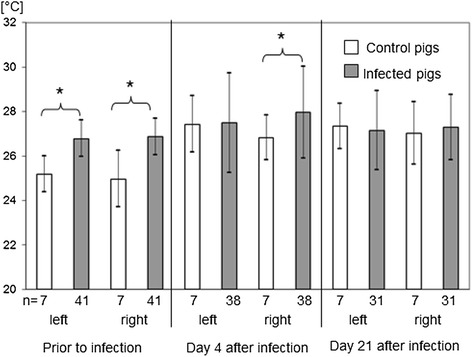
**Absolute skin surface temperatures of the left and right thorax at different stages of infection.** Asterics indicated significant differences between groups (p ≤ 0.05). Data of those pigs were evaluated which were measured in an ambient temperature range between 7-14°C.

**Figure 2:**
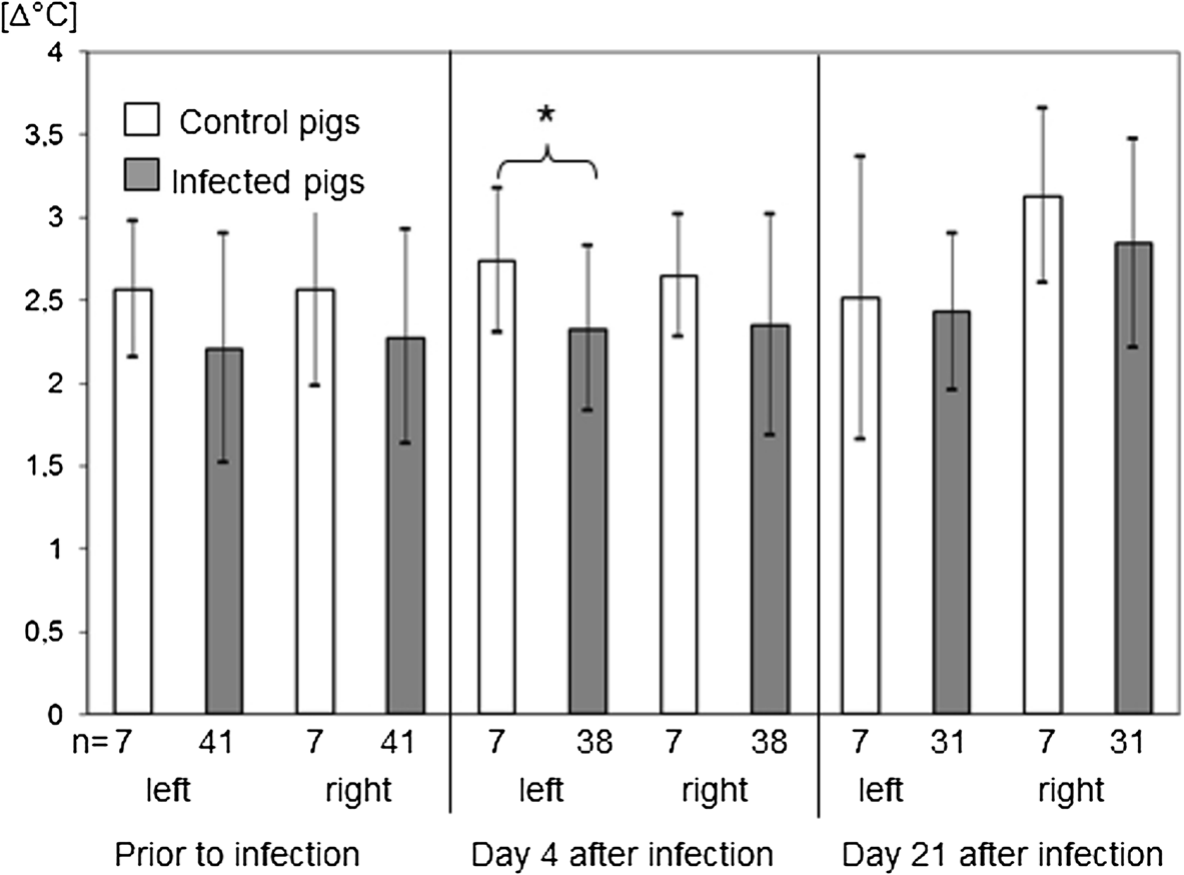
**Temperature differences between thoracal and abdominal ROIs at different stages of infection.** Asterics indicated significant differences between groups (p ≤ 0.05). Data of those pigs were evaluated which were measured in an ambient temperature range between 7-14°C.

Student´s *t*-test for independent samples as well as t-tests for paired observations for the comparison of different stages of infection were performed with those pigs, which have been examined within an ambient temperature range of 7-14°C on all examination days (Figures 1 and 2). A significant difference between infected pigs and control pigs was found for the left skin surface difference temperature between lung ROI and abdominal ROI.

Differences in absolute skin surface temperatures between control pigs and infected pigs were significant already prior to infection (Figure 1), which might be due to the described order of examination. Control pigs were examined always at first in a relatively cool environment to avoid infection. A significant correlation was found between ambient temperature and thoracal skin surface temperatures (p < 0.01).

This obvious influence of ambient temperature onto the thoracal skin surface temperature was statistically corrected in an analysis of covariance. The corrected data revealed diagnostic significance for the skin surface temperature difference between abdominal and thoracal region on the left body site in the height of the 10th thoracic vertebrae (p < 0.01) during the acute stage of infection

As a result of this study, potential cut-offs were suggested, which might allow a practical application of IRT by a specific preselection of pigs for further diagnostics although the method is influenced by ambient temperature and other internal factors.

**Figure 3:**
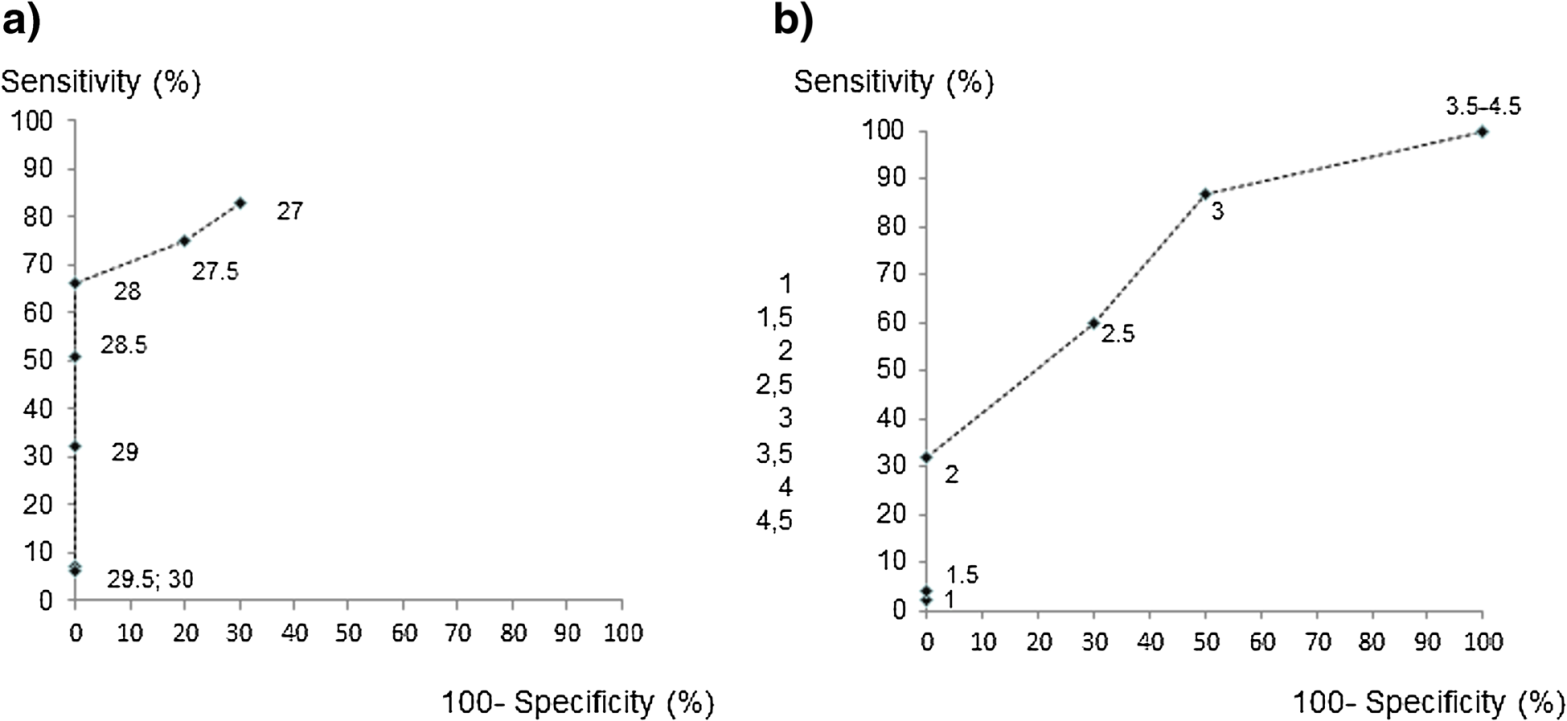
**a, b: Receiver Operating Characteristics (ROC) –curves in the acute stage of infection.** **(a)** absolute skin surface temperature of a thoracal region in the height of the 7th thoracic vertebrae on the right body site (cut-off values in °C). **(b)** the difference between abdominal and thoracal skin temperatures on the left body site (cut-off values in Δ°C). For every cut-off value the 100-specificity on the x-axe and the sensitivity on the y-axe is shown.

During the acute stage of infection a cut off of 28°C of absolute right thoracal temperature resulted in a specificity of the method of 100% (95% confidence interval 69-100%) and a sensitivity of 66% (95% confidence interval 51-79%) as shown in Figure 3a. A cut off of 2°C temperature difference between thoracal and abdominal region on the left body site resulted in a specificity of 100% (95% confidence interval 69-100%) and a sensitivity of 32% (95% confidence interval 19-47%) (Figure 3b).

Maximal abdominal temperatures continuously increased with the days of examination in parallel to an increase in the body weight.

At the chronic stage of infection the lung lesion score was negatively correlated to body weight (r_p_ = −0.5, p < 0.01) and positively correlated to the clinical (r_p_ = 0.5, p < 0.01) and the CT score (r_p_ = 0.8, p < 0.01). No correlations were found between lung lesion and CT scores and the temperature parameters measured by IRT.

### Validity of lung biopsy

*A.pp*. could be reisolated from lung biopsies in twelve out of 50 infected pigs. While the overall *A.pp.* detection rate using this method in comparison to positive lung tissue samples was 19-51% (CI 95%), all ten pigs with severe clinical symptoms, which had been sampled until day 4 after infection, had positive findings for *A. pp.* in bioptates (69-100%, CI 95%).

**Figure 4:**
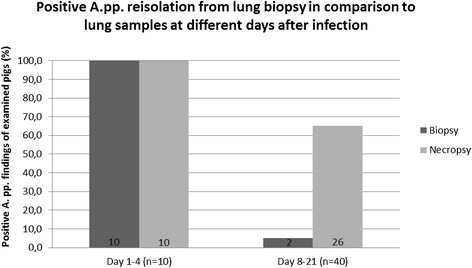
**Comparison of** ***A.pp.*** **reisolation from lung tissue bioptates and lung samples from necropsied pigs.**

Only two animals belonging to the four pigs with the highest clinical score and showing also high lung lesion scores were *A.pp*.-positive in lung biopsy samples on day 21 after infection. Lung tissue samples of the respective pigs were also highly positive. Lung tissue samples of twenty-seven pigs were highly positive for *A.pp*., while in five pigs only moderate and in four pigs only low *A.pp.*-isolation rates were found (Figure 4). *A.pp.* was not reisolated from lung tissue of 14 pigs. A histological examination of lung tissue confirmed pneumonia and pleuritis in 44 out of 50 infected pigs (88%).

## Discussion

The aim of this study was to evaluate IRT as an imaging technique for the diagnostic of respiratory disease in swine. During the trial in living pigs the diagnostic potential of IRT was controlled by CT examination.

Planning of the study was directed by preliminary findings supporting the hypothesis, that gross pathological lung lesions can be successfully detected in pigs by IRT and that the difference temperature between lung region and abdominal region is smaller in pigs with lung alterations due to an inflammatory increase in lung temperature [17]. Optimal ROIs for IRT on the pig thorax had been previously specified by measurement of tissue layer thicknesses in various anatomical positions (data not shown).

In general, IRT is a simple and non-invasive method to assess the surface temperature distribution of the body [18]. An increase in temperature is a basic physical reaction towards a harmful stimulus and is one sign of inflammation besides swelling, pain and redness.

Also under physiological conditions skin temperature - as the outer contact organ of the body with the environment - is influenced by many external and internal factors, e.g. sweat evaporation, vascular perfusion, local tissue metabolism and ambient temperature [19,20]. Anyhow, infrared thermographical images captured under controlled conditions might be interpretable to diagnose specific pathological conditions and might be a helpful tool to monitor specific body reactions towards stress, especially thermal stress [21]. A satisfying reproducibility of IRT in a temperature-controlled environment has been shown previously [21,22].

As hypothezised, mean absolute surface temperatures of the thorax were rising after aerosol challenge during the acute stage of infection (day 4), when highest values were recorded. This temperature increase reflects inflammatory mechanisms in the lung (influx of exsudates, immune cell migration into tissue) as well as systemic reactions (increased body temperature, heart rate, changed respiratory rate). In contrast to that, prior to infection, absolute surface temperatures were lower. Surface temperatures during the chronic stage of infection were slightly higher than those measured prior to infection but lower than in the acute stage of infection. The chronic stage of *A.pp*.-infection is characterized by sequestrated necrotic lung tissue, which is surrounded by a fibrotic capsule. In these altered lung areas neither tissue ventilation, nor blood circulation occur. Obviously these chronic inflammatory processes led not to an increased tissue blood perfusion accompanied by an increase in temperature which can be detected from the outside by IRT. Local temperatures in lung tissue sequesters might be lower than in non-affected lung areas.

Using difference temperatures (Δϑ) between the lung ROI and the abdominal ROI as an allocation base for the detection of inflammatory lung disorders, sensitivity and specificity of the method should be further improved for routine measurements under practical conditions. The hypothesis was that Δϑ was highest prior to infection, due to physiological ventilation of lung tissue with cool air. During the acute stage of infection, when lung tissue was heated due to inflammation, Δϑ was supposed to decrease. In contrast to that, in patient animals Δϑ was not significantly different between the examination prior to infection and after infection. A significant difference was found between day 4 and 21 after infection with a higher difference temperature in the chronic stage of infection.

The high significant difference between lower absolute right lung ROI temperatures prior to infection in comparison to higher temperatures on day 21 after infection was found in all group constellations of patients. In parallel a higher difference temperature between lung and abdominal ROI was observed on day 21 after infection, which was contradictory to the hypothesis. This might be due to a steeper temperature increase of the abdominal ROIs on both body sites, which was obviously not proportional to a temperature increase of the lung ROIs.

The statistical comparison of patient animals with control animals showed, that infected pigs have disease-dependent thoracal surface temperature changes during the acute stage of infection, while no difference was found between both groups on day 21 after infection.

In the chronic stage of infection, three weeks later, abdominal ROI temperature significantly increased from day 4 to day 21 after infection in control pigs and to a much lesser extent in the patient pigs. Briefly, in healthy pigs a higher growth-dependent increase in abdominal than in lung temperature can be assumed. Additionally, differences in abdominal temperatures might be also due to differences in digestive activities. Feed depriviation before examination was at least 12 hours in all animals. Nevertheless, severity of disease affected the appetite of the pigs, so that individual differences in feed consumption occurred during the trial. Some animals still had intestinal contents during examination. The localization of intestine and liver tissue might also be an influencing factor onto thoracal temperatures. The left lung ROI was located at the height of the 10th vertebra where liver tissue was underlying. Filling of the stomach can dislocate the liver in a more cranial position close to the lung. Liver tissue is known to be approximately 1-2°C warmer than the body core temperature [23].

The chronological order of the examination of piglets is assumed to be also a factor influencing the thoracal surface. To avoid contamination of the healthy control pigs with *A.pp*., they were always examined at first. For this reason the cooled chamber was warmer when patients were examined (ambient temperature during examination of control pigs: 8.47 ± 1.57°C and of infected pigs: 11.97 ± 1.23°C). As previously published, ambient temperature strongly affects the results of IRT measurements, although optimal ambient temperatures for IRT have been reported to be <18°C, which was realized in this study [24].

Not only the effect of ambient temperature on IRT measurement results might limit the application of this method on farms, but also the relative long time until an adequate IRT picture can be produced (approximately 30 seconds in anaesthetized pigs). Anatomical landmarks should be visible in the picture, so that the correct ROIs can be positioned for later temperature evaluation. Non-anaesthetized pigs are moving continuously, which will impair the quality of pictures. For this reason further technical improvement of IRT cameras will be necessary to pave the way for this technique for practical usage.

For the use of IRT adequate facilities and a profound knowledge of the anatomy and physiology of the examined species and the pathophysiology of the disease which should be examined might be important to avoid misdiagnosis. Because mostly several different bacterial and viral pathogens are involved in porcine respiratory diseases, also lung alterations will be a combination of different gross pathological findings. A detection of these inconsistent inflammatory findings might be even more difficult than those findings reported after experimental mono-infection in this study. Also under standardized external conditions, IRT is error-prone due to several influencing endogenous factors whose impact cannot adequately be corrected. IRT is not sensitive enough to assess the prevalence of pigs with lung alterations in a herd. But, using a cut off with a high specificity as suggested in this study, IRT might be a promising tool to detect pigs without clinical symptoms but with lung alterations. Using this 2-step diagnostic approach would enable a more targeted and cost-effective diagnostic of respiratory disease.

Bacteriological examination of lung tissue bioptates of infected pigs resulted in *A.pp.* reisolation in 24% of all animals. All animals with severe clinical symptoms, which were euthanized during the first days after infection, had positive findings for *A. pp.* in bioptates. Only 5% of pigs were detected positive by lung bioptates on day 21 after infection. For this reason, lung biopsy can be assessed to be a diagnostic option for *A. pp.* detection only during the acute stage of infection. Biopsy of the lung is highly invasive and can cause further problems as bleeding and sudden death.

It can be concluded, that the assessment of IRT for the diagnostic of lung alterations requires a comparison of infected and non-infected pigs of the same age and under same ambient temperature conditions. The interpretation of findings is complicated by a non-proportional, partly growth-dependent change in surface temperature of different body regions.

In this study, most appropriate surface temperature parameters for the diagnostic of the lung health status of pigs are the absolute temperature of the right thoracal region in the height of the 7th thoracic vertebra as well as the temperature difference between thoracal and abdominal region on the left body site on the height of the 10th thoracic vertebra.

With a next generation of IRT cameras both localizations should be further evaluated for their diagnostic significance in non-anaesthetized piglets with and without lung diseases and under moderate ambient temperature conditions.

## Conclusion

Using IRT lung alternations are better detectable in the acute stage of infection 4 days after infection than in the chronic stage of infection 21 days after infection. The method is interfering with external and internal factors which have to be regarded during the examination and analysis. Especially the effect of ambient temperature on skin surface temperatures visualized in IR-images always has to be considered. The assessment of IRT requires a comparison of infected and non-infected pigs of the same age and under same ambient temperature conditions. The application of this tool in the field might be restricted to a selection of pigs for further diagnostic with adequate specificity.

## Methods

### Animals

A total of 60 clinically healthy male castrated pigs (German Landrace) in the age of four weeks were used in this study. All pigs were bred and raised in a closed breeding herd of high health status that is routinely tested negative for *A. pp.*, Porcine Reproductive and Respiratory Syndrome Virus (PRRSV), toxigenic *Pasteurella multocida,* endo- and ectoparasites.

To guarantee the health status of the pigs, all pigs used in this study were checked for antibodies against *A. pp.* using an Apx-II-Enzyme-linked immunosorbant assay (ELISA) after arrival [25]. Animals were housed and cared under standardized conditions according to the Directive of the European Convention for the Protection of Vertebrae Animals Used for Experimental and Other Scientific Purposes (European Treaty Series, nos. 123 [http://conventions.coe.int/treaty/EN/treaties/html/123.htm] and 170 [http://conventions.coe.int/treaty/EN/treaties/html/170.htm]) at the Institute for Microbiology, University of Veterinary Medicine Hannover, Germany. The study was approved from the local permitting authorities in the Lower Saxony State Office for Consumer Protection and Food Safety and in accordance with the requirements of the national animal welfare law (approval number: 33.9-42502-12/0835). Precautions aimed at avoiding unnecessary suffering were taken at all stages of the experiment.

Animals were randomised in two control groups of five and five challenge groups of ten.

Twice a day (8 am and 5 pm) a commercial weaner pig diet (Deuka Primo, Deutsche Tiernahrung Cremer GmbH & Co. KG, Bremen, Germany) was fed with exception of the examination days and the day of infection, when pigs were fed in the evening after the manipulations, to lower the risk of a circuit failure under anaesthesia and to minimize the effect of digestion onto the body surface temperature. Water was given ad libitum.

### Experimental setup

Because of the high number of animals, which could not be examined within one trial due to logistic reasons, two sequential trials following the same protocol during the examination period of 28 days were performed. In the first trial 30 animals and in the second trial 20 animals were challenged with *A. pp.,* while 5 pigs served as a mock-infected control group during each trial. In total, in this study data of 50 pigs challenged with *A.pp.* and 10 control pigs were evaluated. A clinical examination of all pigs with special emphasis onto gastrointestinal and respiratory signs, locomotory disorders, skin alterations and behaviour was performed daily in all pigs resulting in daily clinical scores. The first CT and IRT examination as well as blood sampling were performed in the first week, when all pigs had been assessed as healthy.

As soon as deep anaesthesia was achieved by 15 mg ketamine (Ursotamin®, Serumwerk-Bernburg AG, Bernburg, Germany)/kg body weight (bw) intramuscularly (i.m.) and 2 mg azaperon (Stresnil®, Janssen-Cilag GmbH, Baar, Switzerland) /kg bw i.m., pigs were at first examined by computed tomography followed by a cooling down of 15 minutes in a cooling chamber (6-15°C) and then IRT examination of the thorax. Subsequently pigs were brought back to their stables and monitored until they awoke. A few days after the first examinations, aerosol challenge was performed on day 0 of the infection experiment.

The second examination was performed according to the same protocol on the fourth day after aerosol challenge during the acute stage of infection and the third examination on day 21 after infection during the chronic stage of infection. After the last examination, lung biopsies were sampled under deep anaesthesia and pigs were then euthanized (60 mg pentobarbital (Euthadorm®, CP-Pharma, Burgdorf, Germany)/ kg bw intravenously) and necropsied to assess gross pathological lung alterations and to take further diagnostic samples.

### Aerosol challenge

Aerosol challenge was performed as previously described [26,27] using an *A.pp.* bioptype 1 serotype 2 strain for infection, which has been isolated from a naturally infected pig in Germany (lab identification number C3656/0271/11, Institute of Microbiology, University of Veterinary Medicine, Hannover, Germany).

Briefly, groups of five pigs were infected simultaneously in an aerosol chamber. Within 2 minutes at a pressure of 2 bar 13 ml of the diluted culture (containing 3 × 10^7^ – 8 × 10^9^ bacteria) were nebulized in the chamber resulting in approximately 1 × 10^2^ colony forming units (cfu) of *A.pp.* per litre aerosol. Subsequently pigs were exposed for 10 minutes to the aerosol in the closed chamber. Challenge doses were confirmed retrospectively by overnight culture of several dilutions of the bacterial inoculum and determination of the cfu.

For the mock challenge of control pigs 13 ml of a 154 mM sterile NaCl solution was nebulized in the aerosol chamber.

### Clinical examination

Clinical scoring and the classification of disease severity were performed according to Hoeltig et al. [28] with the exception of pulse oxymetry. Animals were monitored daily for clinical signs of disease assessing the following parameters: breathing noise, type of respiration, breathing rate, coughing, skin colour, posture, behaviour, feed intake, body temperature, and symptoms of gastrointestinal disorders as vomiting or diarrhoea. Cumulative clinical scores were calculated for each pig as a sum of daily clinical scores from day of arrival to infection (day 0), from day 0 to 4 and from day 0 to 21. Disease classifications by clinical scores on day 0 and 4 were: not affected (0–0.70), slightly affected (0.71-7.13), moderately affected (7.14-13.56) and severely affected (>13.56). Disease classifications by clinical scores on day 21 were: not affected (0–2.00), slightly affected (2.01-34.7), moderately affected (34.71-67.3) and severely affected (>67.3).

### CT examination

CT examination of the pig thorax has been described in detail by Brauer et al. [6]. Briefly, anaesthezised pigs were positioned symmetrically in sternal recumbency. From the cranial thoracic aperture to the caudal end of the lung the thorax was scanned by a third generation single-slice CT scanner (Philips Tomoscan M, Philips Medical Systems, Germany) with defined settings for scanogram and volume scan (tube voltage 120 kV, current 40 mA, slice thickness 7 mm, reconstruction interval 5 mm, pitch 1.5). CT scores (CTS) for each animal were calculated as previously described by Brauer et al. [6] taking morphological alterations and their distribution pattern, as well as absorption densities into account. Disease classification by CTS was: not affected (0–0.23), slightly affected (0.24-1.70), moderately affected (1.71-2.22) and severely affected (>2.23).

### Infrared thermographical examination

Anaesthetized pigs were positioned in sternal recumbency on a plastic tray and metal markers were fixed at the 5th, 7th, 10th and 13th thoracic vertebrae left and right beside the spine to serve as anatomical landmarks. Pigs were left for acclimatization in a cooling chamber (average ambient temperature of 11.27 ± 2.29°C) for 15 minutes. Prior to and after this acclimatisation period heart frequency, respiratory rate, and body temperature were recorded. IRT images were made from the left and right side of the body in a constant distance, right-angled to the thorax with an IR camera (VarioCAM hr Inspec, Infratec, Dresden, Germany) after the acclimatization period. Presetting of the camera was a premium mode with a camera-internal repeated calibration prior to IRT imaging [11]. Thus, a continuous balancing of inhomogeneities in the microbolometer array was possible. An emissivity of 0.96, which is a good assumption for skin with little hair, was presetted [29]. Picture matrix was 384 x 288 pixels and the thermical resolution was 50 mK. Additionally, a reference body with constant room temperature placed next to the pig was recorded on each image as an internal standard. Room temperature was recorded prior to every single examination. For analysis of IR images a standard Windows PC and an image analysis software package (Irbis3, InfraTec GmbH, Dresden, Germany) were used.

Two certain ROIs, one on the right side of the thorax in the height of the 7th thoracic vertebra (R7vLr) and one on the left side of the thorax in the height of the 10th thoracic vertebra (R10vLl) were chosen as measurement localizations, because outer tissue layers (skin, fat, muscle) were thin at these anatomical positions (data not shown). These two ROIs were selected to evaluate thoracal surface temperatures in each pig at three examination days: prior to infection, when pigs were healthy, on day 4 after infection during the acute stage of disease and on day 21 after infection during the chronic stage of infection, when pigs did not show respiratory clinical signs any longer.

To transform real anatomical positions into two dimensional IR images, visible markers were positioned onto the pig. These metal markers served as anatomical landmarks and specific orientation points (OP) to reconstruct the exact positions of the 5th, 7th, 10th or 13th thoracic vertebra (TV) in the IR images.

**Figure 5:**
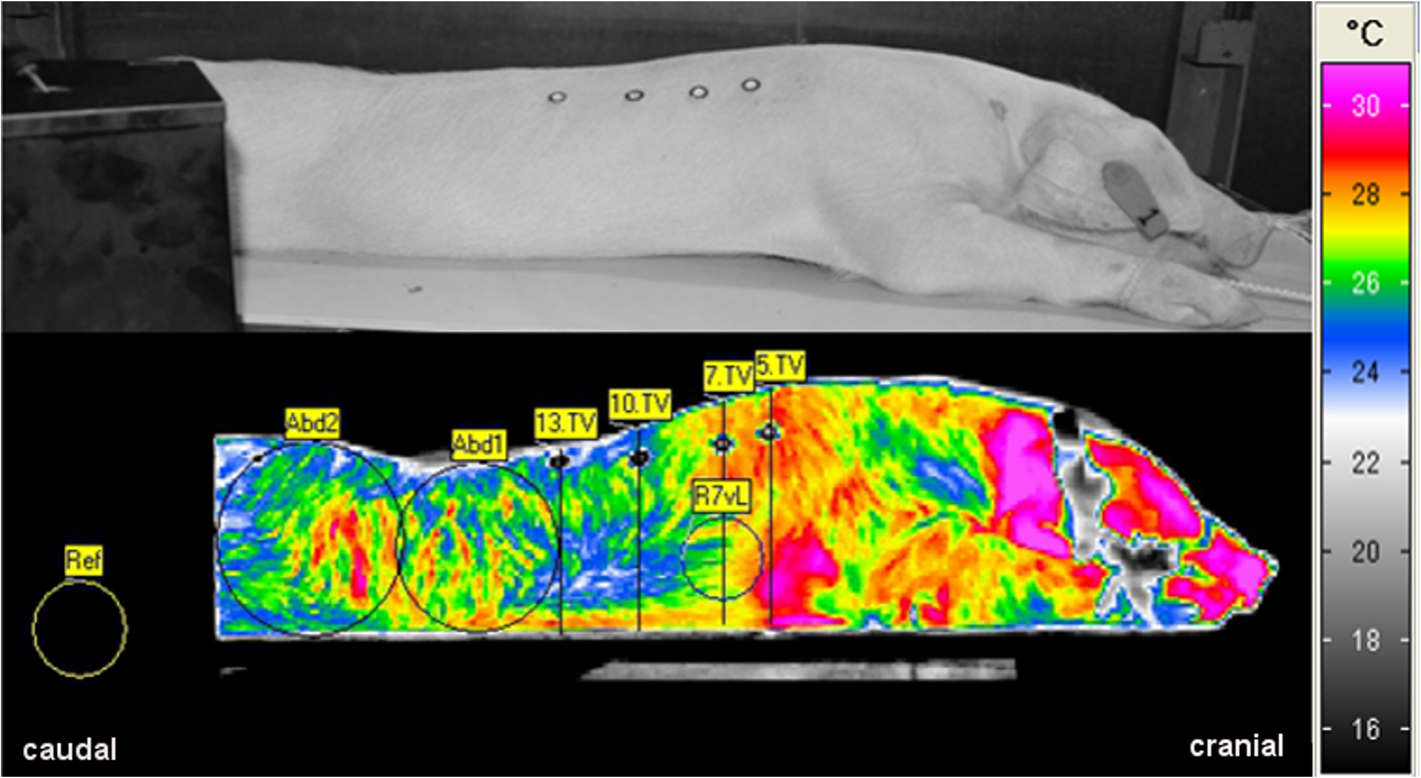
**IR image of a highly infected pig in sternal recumbency with ROIs and reference lines.** Day 4 after infection, right view. T_A_ = 13.6°C, colour range: SW-VarioCam, temperature range = 23–34°C, picture matrix: 384 x 288 pixel, emissivity of 0.96. 5.TV = vertical line of the 5th thoracic vertebra (TV), 7.TV = vertical line of the 7.TV,10.TV = vertical line of the 10.TV, 13.TV = vertical line of the 13.TV, Ref = ROI of the reference body, Abd1 and Abd2 = abdominal ROIs, R7vL = lung ROI of the 7.TV.

Two additional ROIs were positioned in the abdominal region in order to cover as much as possible of the abdominal area (Abd_1_, Abd_2_). Abd_1_ touched tangentially the dorsal and ventral bodyline as well as the orientation point in the height of the 13th thoracic vertebra (OP13). Abd_2_ was positioned adjacent to Abd_1_ tangentially to the dorsal and ventral bodyline covering the remaining caudal part of the abdomen (Figure 5). Maximal abdominal surface temperatures (ϑ_Max)_ and standard deviations were recorded. The ROI of the reference body (Ref) with a radius of 18 pixels was representative for the ambient temperature measured onto the surface of a black reference body.

Mean (ϑ_M_) temperature of the lung ROIs and the ROI of the reference body were documented.

Difference temperatures between mean lung ROI temperature and maximal abdominal ROI temperatures (Abd_1_ or Abd_2_) were calculated according to the following equation.

$$ \Delta \upvartheta \mathrm{R}\mathrm{x} = {\upvartheta}_{\mathrm{maxAbd}1,\ \mathrm{A}\mathrm{b}\mathrm{d}2}-{\upvartheta}_{\mathrm{meanRx}} $$

Measured absolute skin surface temperatures were corrected by the measurement error determined using the reference body as an allocation base.

### Pathological, bacteriological and histological examination

Prior to euthanasia, those areas on the thoracal surface, which were obviously warmer than the surrounding tissue, were detected immediately by IRT imaging and directed lung biopsy was performed. If no warm areas could be detected by eye, lung biopsy was performed on the right side of the thorax between the fifth and the seventh rib. Lung tissue bioptates were divided and provided for histological and microbiological examinations.

After lung biopsy, control pigs and infected pigs were euthanized and necropsied. Macroscopic lung alterations were quantified according to the Lung Lesion Score (LLS) proposed by Hannan et al. [30] and specified in the European Pharmacopoeia for vaccine development [31]. Briefly, lung tissue alterations assessed by visual inspection and palpation were recorded in a schematic map of the porcine lung consisting of 74 triangles (7 triangles for cardial and apical lobes each, 8 triangles for the accessory lobe, 19 triangles for each of the diaphragmatic lobes). The quotient of altered triangles and whole triangles of each lobe was multiplied by 5, so that each lobe could reach a maximum score of five and the whole lung a maximum lung lesion score of 35. The classification of the score was: not affected (0), slightly affected (0.1-5.0), moderately affected (5.1-10.0) and severely affected (>10.0). In addition, a score according to the Slaughterhouse Pleurisy Evaluation System (S.P.E.S.) was used, which allows a rapid and efficient quantification of pleuritis [32].

Altered lung tissue as well as lung bioptates were cultivated on selective meat and blood agar to reisolate *A.pp*. or to confirm, that control pigs stayed *A.pp*.-negative until the end of the study [33]. Bacterial species diagnostic was confirmed by PCR for the ApxIIA gene and by urease activity [34,35].

### Statistical analysis

Statistical analyses of data were performed using SAS® software, Version 9.3 (SAS Institute Inc., Cary, NC, USA). Right and left thoracal temperatures as well as the difference temperatures between thoracal and abdominal regions on the right and left body site were compared using student`s *t*-test for independent samples (2-sample-*t*-test) for group comparisons and *t*-test for dependent samples (1-sample-*t*-test for differences) for paired samples during the course of infection. Data were normally distributed. The Pearson´s correlation coefficients were calculated between these four dependent variables and various parameters as shown in Tables 2 and 3. The Spearman´s correlation coefficient was calculated for the S.P.E.S. and LLS scores. Multiple regression analysis was performed for the four diagnostic parameters, absolute left and right thoracal surface temperatures and left and right difference temperatures between thoracal and abdominal surfaces with various influencing variables as body weight, ambient temperature, body core temperature, right and left abdominal temperature, heart rate and breathing frequency (Tables 2 and 3). An analysis of covariance was performed to correct for the influence of ambient temperature onto the targeted variables.

## Abbreviations

*A.pp.*: *Actinobacillus pleuropneumoniae*
IRT: Infrared thermography
CT: Computed tomography
ROI: Region of interest
ELISA: Enzyme-linked immunosorbant assays
CLS: Clinical score
CTS: Computed tomography score
LLS: Lung lesion score
Cl: Confidence interval
PRRSV: Porcine Reproductive and Respiratory Syndrome Virus
bw: Body weight
i.m.: Intramuscularly
OP: Orientation point
TV: Thoracic vertebra
Abd_1_,Abd_2_: Abdominal ROIs
R10vLl: ROI of the 10.TV on the left side of the thorax
R7vLr: ROI of the 7.TV on the right side of the thorax
S.P.E.S.: Slaughterhouse Pleurisy Evaluation System

## References

[1] Opriessnig T, Giménez-Lirola LG, Halbur PG (2011). Polymicrobial respiratory disease in pigs. Anim Health Res Rev.

[2] Hansen MS, Pors SE, Jensen HE, Bille-Hansen V, Bisgaard M, Flachs EM, Nielsen OL (2010). An Investigation of the pathology and pathogens associated with porcine respiratory disease complex in Denmark. J Comp Pathol.

[3] Hensel A, Ganter M, Kipper S, Krehon S, Wittenbrink MM, Petzoldt K (1994). Prevalence of aerobic bacteria in bronchoalveolar lavage fluids from healthy pigs. Am J Vet Res.

[4] Sorensen V, Jorsal SE, Mousing J, Straw BE, Zimmerman JJ, Allaire SD, Taylor DJ (2006). Diseases of the respiratory system. Diseases of Swine.

[5] Brauer C, Hoeltig D, Hennig-Pauka I, Beyerbach M, Gasse H, Hewicker-Trautwein M, Gerlach GF, Waldmann KH (2011). Computed tomography of the pig lung: an innovative approach to the definition of the pulmonary health status. Tierärztl Prax G.

[6] Brauer C, Hennig-Pauka I, Hoeltig D, Buettner FF, Beyerbach M, Gasse H, Gerlach GF, Waldmann KH: **Experimental*****Actinobacillus pleuropneumoniae*****challenge in swine: Comparison of computed tomographic and radiographic findings during disease.** *BMC Vet Res* 2012, **8**. online publication: doi:10.1186/1746-6148-8-47. 10.1186/1746-6148-8-47 PMC3537595 22546414

[7] Heinritzi K, Beisl J (1995). Untersuchungen zur Verwendbarkeit der Sonographie beim Schwein. Dtsch tierärztl Wschr.

[8] Hoeltig D, Hennig-Pauka I, Beyerbach M, Thies K, Rehm T, Gerlach GF, Waldmann KH, FUGATO-consortium IRAS (2008). Comparison of the diagnostic significance of clinical, radiographic and ultrasonographic results after an experimental aerosol infection of pigs with *Actinobacillus pleuropneumoniae*. Berl Munch Tierarztl Wochenschr.

[9] Reinhold P, Rabeling B, Guenther H, Schimmel D (2002). Comparative evaluation of ultrasonography and lung function testing with the clinical sings and pathology of calves inoculated experimentally with *Pasteurella multocida*. Vet Rec.

[10] Siewert C, Dänicke S, Kersten S, Brosig B, Rohweder D, Beyerbach M, Seifert H (2014). Difference method for analysing infrared images in pigs with elevated body temperatures. Z Med Phys.

[11] Infratec GmbH (2009). Benutzerhandbuch VarioCAM high resolution, Stand.

[12] Rich PB, Dulabon GR, Douillet CD, Listwa TM, Robinson WP, Zarzaur BL, Pearlstein R, Katz LM (2004). Infrared thermography: a rapid, portable, and accurate technique to detect experimental pneumothorax. J Surg Res.

[13] Schweinitz D (1999). Thermographic diagnostics in equine back pain. Vet Clin North Am Equine Pract.

[14] Tunley BV, Henson FM (2004). Reliability and repeatability of thermographic examination and the normal thermographic image of the thoracolumbar region in the horse. Equine Vet J.

[15] Schaefer AL, Cook NJ, Church JS, Basarab J, Perry B, Miller C, Tong AK (2007). The use of infrared thermography as an early indicator of bovine respiratory disease complex in calves. Res Vet Sci.

[16] Savary P, Hauser R, Osse P, Jungbluth T, Gygax L, Wechsler B (2008). Eignung der Thermographie zur Erfassung von Entzündungen an den Gliedmaßen von Mastschweinen. Dtsch tierärztlWschr.

[17] Siewert C, Hoeltig D, Brauer C, Seifert H, Hennig-Pauka I: **Medical infrared imaging of the porcine thorax for diagnosis of lung pathologies.** In *Proceedings of the 21th International Pig Veterinary Society Congress 2010 Vancouver.* Edited by D’Allaire S, Friendship R. Vancoiuver: 663. Volume II.

[18] Fikackova H, Ekberg E (2004). Can infrared thermography be a diagnostic tool for arthralgia of the temporomandibular joint?. Oral Surg Oral Med Oral Pathol Oral Radiol Endod.

[19] So YT, Aminoff MJ, Olney RK (1989). The role of thermography in the evaluation of lumbosacral radiculopathy. Neurology.

[20] Kateb B, Yamamoto V, Yu C, Grundfest W, Gruen JP (2009). Infrared thermal imaging: a review of the literature and case report. Neuroimage.

[21] Zaproudina N, Varmavuo V, Airaksinen O, Naerhi M (2008). Reproducibility of infrared thermography measurements in healthy individuals. Physiol Meas.

[22] Huygen FJ, Niehof S, Klein J, Zijlstra FJ (2004). Computer-assisted skin videothermography is a highly sensitive quality tool in the diagnosis and monitoring of complex regional pain syndrome type I. Eur J Appl Physiol.

[23] Henssge C, Madea B, Benecke M, Berg S, Geyh MA, Karger B, Krause D, Lignitz E, Rothschild MA, Brinkmann B, Madea B (2004). Leichenerscheinungen und Todeszeitbestimmung. Handbuch gerichtliche Medizin.

[24] Roehlinger P (1979). Zur Anwendung der Infrarottechnik in der Veterinärmedizin. Arch Exper Vet med.

[25] Leiner G, Franz B, Strutzberg K, Gerlach GF (1999). A novel enzyme-linked immunosorbent assay using the recombinant *Actinobacillus pleuropneumoniae* ApxII antigen for diagnosis of pleuropneumonia in pig herds. Clin Diagn Lab Immunol.

[26] Jacobsen M, Nielsen J, Nielsen R (1996). Comparison of virulence of different *Actinobacillus pleuropneumoniae* serotypes and biotypes using an aerosol infection model. Vet Microbiol.

[27] Maas A, Jacobsen ID, Meens J, Gerlach GF (2006). Use of an *Actinobacillus pleuropneumoniae* multiple mutant as a vaccine that allows differentiation of vaccinated and infected animals. Infect Immun.

[28] Hoeltig D, Hennig-Pauka I, Thies K, Rehm T, Beyerbach M, Strutzberg-Minder K, Gerlach GF, Waldmann KH, FUGATO-consortium IRAS (2009). A novel Respiratory Health Score (RHS) supports a role of acute lung damage and pig breed in the course of an *Actinobacillus pleuropneumoniae* infection. BMC Vet Res.

[29] Diakides NA, Bronzino JD (2008). Medical Infrared Imaging.

[30] Hannan PC, Bhogal BS, Fish JP (1982). Tylosin tartrate and tiamutilin effects on experimental piglet pneumonia induced with pneumonic pig lung homogenate containing mycoplasmas, bacteria and viruses. Res Vet Sci.

[31] European Pharmacopoeia (Ph. Eur.): **Porcine actinobacillosis vaccine (inactivated).** In *European Pharmacopoeia 7th edition.* 2008:926–927. Section: Vaccines.

[32] Dottori M, Nigrelli AD, Bonilauri P, Merialdi G, Gozio S, Cominotti F (2007). Proposta per un nuovo sistema di punteggiatura delle pleuriti suine in sede di macellazione: La griglia SPES (Slaughterhouse Pleurisy Evaluation System). Large Anim Rev.

[33] Jacobsen MJ, Nielsen JP (1995). Developement and evaluation of a selective and indicative medium for isolation of *Actinobacillus pleuropneumoniae*. Vet Microbiol.

[34] Buettner FFR, Bendallah IM, Bosse JT, Dreckmann K, Nash JHE, Langford PR, Gerlach GF (2008). Analysis of the *Actinobacillus pleuropneumoniae* ArcA regulon identifies fumarate reductase as a determinant of virulence. Infect Immun.

[35] Maas A, Meens J, Baltes N, Hennig-Pauka I, Gerlach GF (2006). Development of a DIVA subunit vaccine against *Actinobacillus pleuropneumoniae* infection. Vaccine.

